# Promiscuity in Nature Extends to Central Protein Biosynthetic Machinery

**DOI:** 10.1021/acscentsci.5c00387

**Published:** 2025-03-10

**Authors:** April L. Lukowski

**Affiliations:** † Center for Marine Biotechnology and Biomedicine, Scripps Institution of Oceanography, University of California San Diego, La Jolla, California 92093, United States; ‡ Skaggs School of Pharmacy and Pharmaceutical Sciences, University of California San Diego, La Jolla, California 92093, United States

The process of translation is a core necessity for life, generating proteins that power metabolism from the nucleic acid language of cells. The biochemical reactions that take place as mRNA is decoded and converted into peptides by the ribosome via tRNA units constitute fundamental knowledge today–but how have these processes come to be? Can we catch a glimpse of the biosynthetic processes of prebiotic life by examining what currently exists?^1^

**Figure 1:**
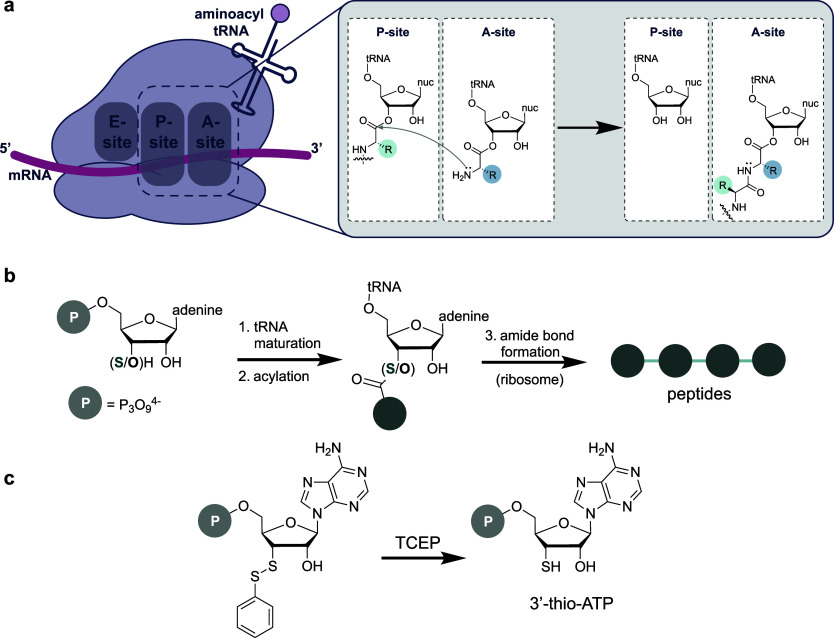
(a) Native ribosomal translation and oxo-ester mechanism. (b) Key steps in translation probed by Kent et al. (c) Synthesized protected thio-ATP analogue and species generated in situ via reduction with tris(2-carboxyethyl)phosphine (TCEP).

Protein synthesis at the ribosome involves the use of oxo-esters to facilitate peptide bond formation (Figure 1a). In stark contrast, peptide bond synthesis in other biosynthetic pathways, such as those used in nonribosomal peptide synthetases (NRPSs), requires the use of thioester intermediates. Thioesters represent a ubiquitous biosynthetic strategy for C–C, C–N, and C–O bond formation in nature, as exhibited by other crucial pathways, such as fatty acid biosynthesis, the citric acid cycle, and polyketide synthase-containing pathways.^2,3^ Thioesters are highly reactive, polarizable, and widely thought to be prevalent in the prebiotic world, thereby representing a possible early strategy for peptide synthesis.^4^ Kent et al. ask whether thioesters can be tolerated by extant protein biosynthetic machinery at several steps in the process: nucleotidyl transfer to truncated tRNAs, aminoacylation of tRNAs, and peptide bond formation in the ribosome (Figure 1b).

To facilitate their studies, the authors synthesized an ATP analogue, 3′-thio-3′-deoxyadenosine triphosphate (3′-thio-ATP) from (+)-xylose in 10 steps, protected with a thiophenol (Figure 1c). The first experiment was to test whether 3′-thio-ATP could be efficiently integrated into a key step of tRNA maturation–appending nucleotides to truncated tRNAs. Using an *Escherichia coli* tRNA nucleotidyltransferase, which is known to accept modified bases but has only demonstrated activity on one other 3′-analogue, the authors used *in vitro* transcription to probe the incorporation of 3′-thio-ATP into different tRNAs lacking adenosine at the terminus. Quantitative conversion was achieved on all three tRNA substrates in the absence of contaminating ATP, which effectively outcompeted the non-native 3′-thio-ATP, demonstrating for the first time that a thioester nucleotide analogue could be tolerated.

Next, Kent et al. assessed the potential for terminally thiolated tRNAs to load natural and unnatural amino acids using both *E. coli* and *Methanomethylophilus alvus* derived aminoacyl tRNA synthetases and laboratory evolved flexizymes,^5^ ribozymes that tolerate large substrate scopes for aminoacylation. Remarkably, all three of the synthetases tested were capable of acylating the 3′-thio-tRNA with both canonical and unnatural amino acids, and the flexizymes were also capable of utilizing the modified tRNA for aminoacylation. In testing a thioester-containing substrate with flexizyme, the authors also noted that thioester exchange proceeded in the absence of flexizyme, demonstrating an enzyme-independent method for generating acylated tRNAs, which models prebiotic hypotheses for peptide synthesis.^4^

In native translation, oxo-esters are known to exchange between the 2′- and 3′-positions, but the behavior of a 3′-thioester in this situation could not be predicted. To evaluate the reactivity of transacylation, the authors synthesized a 2′-acylated version of 3′-thio-ATP and tracked whether the molecule could isomerize by ^13^C NMR and observed no thioester formation. However, when they exposed their mixture to cysteine methyl ester, considerable dipeptide was formed, analogous to a product of native chemical ligation. In comparison to a crucial control using triacylated adenosine as a substrate, it was clear that such products were only able to form in the presence of 3′-thio-ATP.

In the final feat of the study, Kent et al. tested the potential of their acylated thioester tRNAs to support ribosomal translation. By comparing the incorporation of unnatural amino acids with both oxo-ester and thioester tRNAs, the authors could show that the thioester tRNA was still capable of promoting translation, albeit at a reduced rate of incorporation relative to the standard oxo-ester tRNA system. Taken together, the results of this study elegantly show that native translation machinery exhibits promiscuity that may hint at a prior biosynthetic strategy for peptide synthesis.

The question of how protein synthesis arose is one of the greatest puzzles in chemical evolution. Thioesters, being more reactive than oxo-esters, are thought to have promoted an early phosphate-free, enzyme-independent method for peptide synthesis in the prebiotic world.^6^ The result is a world with less catalytic control, in contrast to modern central biochemical processes that are considered to be finely tuned. Perhaps there is a selectivity advantage of utilizing oxo-esters resulting in more control over the translation process we observe today in comparison to a thioester-based mechanism.

From a synthetic biology perspective, expanding the biochemical toolkit for incorporating diverse and unnatural monomers into sequence defined polymers remains a challenge requiring creative solutions.

This foundational study will likely spark inspiration for continued outside-the-box exploration of native biochemical processes.
